# Mobile phone addiction and mental health: the roles of sleep quality and perceived social support

**DOI:** 10.3389/fpsyg.2023.1265400

**Published:** 2023-09-22

**Authors:** Lin-Lin Yang, Chen Guo, Geng-Yin Li, Kai-Peng Gan, Jin-Huan Luo

**Affiliations:** ^1^School of Law and Political Science, Yunnan University of Finance and Economics, Kunming, Yunnan, China; ^2^School of Physics and Electronic Engineering, Yuxi Normal University, Yuxi, Yunnan, China

**Keywords:** mobile phone addiction, sleep quality, perceived social support, mental health, university students

## Abstract

As a global phenomenon, mobile phone addiction has become an increasingly common issue among Chinese university students. Although previous research explored the link between mobile phone addiction and mental health, the possible mechanism underlying the above association is unclear. We administered a cross-sectional survey to 585 participants from two universities in Kunming, southwest China, from October 2021 to January 2022. Our results suggested that mobile phone addiction was negatively associated with mental health, and sleep quality partially mediated the relationship between mobile phone addiction and mental health. Furthermore, perceived social support positively moderated the direct effect of sleep quality on mental health, as well as the indirect effect of mobile phone addiction on mental health. These findings provide a new insight into the underlying mechanism by which mobile phone addiction affects university students’ mental health. The results emphasize a necessary task for administrators, health workers, and family members to attach importance to the overuse of mobile phones among university students.

## 1. Introduction

In recent years, mobile phones have rapidly gained popularity because of the practicability and convenience that these technologies offer. The 49th statistical report on internet development in China revealed that the total number of mobile phone users stood at 1.64 billion as of December 2021. These ubiquitous innovations are increasingly integrated into individuals’ lives, thereby bringing about consequences. Although mobile phones are prevalent for all age groups, adolescents are the most common users (Gangadharan et al., 2022; Huang et al., 2022). Mobile phone overuse among adolescents may be closely related to psychological health problems, including sleep disturbance, technostress, low self-confidence, social isolation, and depression (Jun, 2016; Tao et al., 2017; Mahmoodi et al., 2018; Thomee, 2018; Park et al., 2019). Mobile phone addiction may therefore result in various health-related issues among teenagers and young adults, as recognized by scholars from different disciplines (Thomee, 2018; Dowran, 2020).

**Figure 1:**
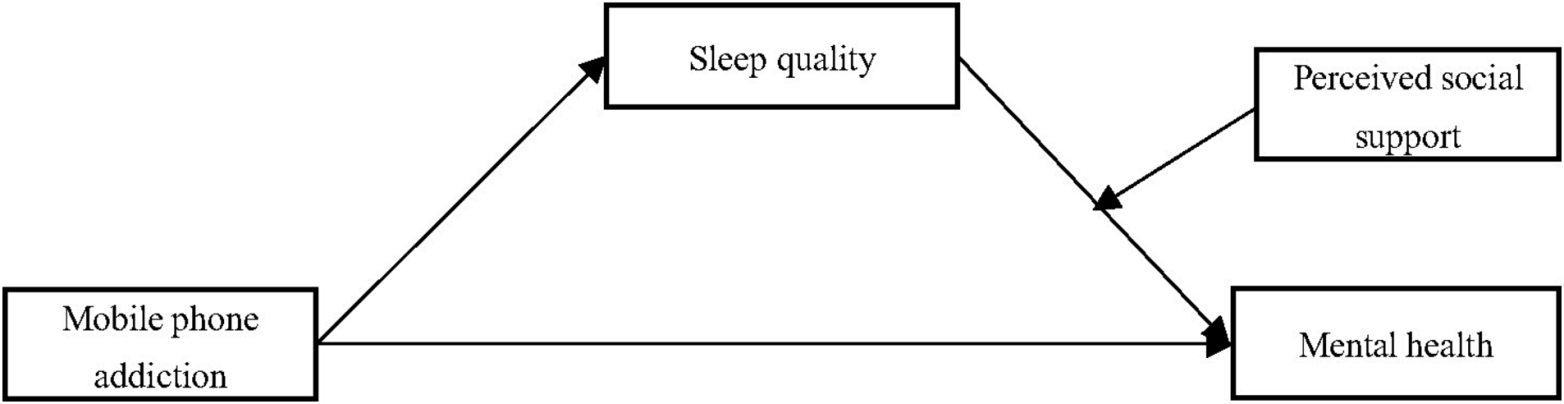
Hypothesized research model.

Although some empirical research has explored the linkage between mobile phone addictions and mental health, the mechanisms by which mobile phone addiction influences adolescents’ mental health have received little attention. That is, scholars have not fully explained the intermediate variables that potentially influence the association between mobile phone addiction and mental health. To date, only a few researches have inquired into possible determinants of the aforementioned association among young adults (Tao et al., 2017; Yang et al., 2019; Ivanova et al., 2020). Therefore, to clarify the contradictions in previously derived findings, the current work examined the effects of mobile phone addiction on mental health on the basis of a sample of college students in China. This study also casts light on the mechanisms that may play a crucial role in such a link by introducing two new intermediate variables, namely sleep quality and perceived social support (Figure 1).

## 2. Literature review and hypotheses

### 2.1. Mobile phone addiction and mental health

Mobile phone addiction, which originated with the development of communication technology, is defined as “a behavioral addiction, characterized by the basic symptoms of addictive behaviors” (Ivanova et al., 2020, p. 656). As a subset of behavioral addiction, it is an impulse control disorder that may have the same outcome as substance use and pathological gambling (Dowran, 2020). Although scholars remain in dispute about whether mobile phone overuse is considered a behavioral addiction (Billieux et al., 2015; Yang et al., 2019), this condition is widely believed to be a kind of uncontrollable and impulsive desire to use mobile phones (Liu et al., 2018; Hao et al., 2019).

The prevalent view is that mobile phone addiction leads to consequences such as psychological health problems, loneliness, depression, and psychiatric disorders (Yang et al., 2019; Dowran, 2020; Augner et al., 2023). Recently, some empirical research has explored the association between mobile phone addiction and mental health (Tao et al., 2017; Mahmoodi et al., 2018; Thomee, 2018; Zhang et al., 2020). A case in point is Seo et al. (2016), who confirmed the close association between mobile phone dependence and adolescents’ negative moods. This finding is supported by more recent empirical evidence that mobile phone overuse significantly and negatively affects adolescents’ psychological problems, such as loneliness, mental health, depression, and social anxiety (Elhai et al., 2017; Calpbinici and Tas Arslan, 2019; Li et al., 2020; Zhang et al., 2020). Furthermore, the findings by Zhang et al. (2020) also indicated that mobile phone dependence is closely related to individual adjustment and mental health status. Based on these results, we formulated the following hypothesis:

*Hypothesis 1*: Mobile phone addiction is negatively associated with mental health.

### 2.2. The mediating effect of sleep quality

Sleep quality can be defined as “difficulty falling asleep and/or maintaining sleep” (Buysse et al., 1989, p. 1), a definition that was reframed by Yi et al. (2006, p. 3) into “the degree of excellence in sleep.” On the basis of the resource model of self-control (Baumeister et al., 1994), when individuals’ psychological resources are in a state of self-depletion, they fail to control themselves. The self-regulatory resources of individuals are diminished by sleep deprivation, thereby stimulating potentially risky behaviors (Barnes et al., 2011; Christian and Ellis, 2011). Consequently, sleep-deprived individuals are more strongly predisposed than their well-rested counterparts to addiction to the internet or mobile phones and even suffer from a high risk of mental health problems (Reis et al., 2019, 2021). Previous empirical research confirmed that individuals with poor sleep quality are more vulnerable to mental diseases than others with good sleep quality (Al-Khani et al., 2019; Suna and Ayaz, 2022). To date, scholars have agreed that poor sleep quality remarkably brings about low levels of mental well-being among university students (Ghrouz et al., 2019; Clement-Carbonell et al., 2021; Cramm et al., 2021).

Poor sleep quality is reported to be closely related to mobile phone addiction (Shin et al., 2017; Friedrich and Schlarb, 2018; Cheng and Meng, 2021). Some empirical research, such as those carried out by Adams et al. (2013) and Kang et al. (2020) have shown that human melatonin secretion will be inhibited if individuals are chronically exposed to the light emitted by mobile phones, and then impairs sleep quality. Put differently, mobile phone overuse may disturb the sleep of individuals and delay the interval within which they fall asleep (He et al., 2020). These results find support in other empirical investigations (Körmendi et al., 2016; Bartel et al., 2019; Haripriya et al., 2019; Sanusi et al., 2022; Zhang et al., 2022), such as that of Haripriya et al. (2019), who demonstrated that young adults have minimal sleep times and potential sleep disorders because they are addicted to social networking and information sharing through mobile phones at night. This evidence is extended by the latest studies, which corroborated that mobile phone addiction has significant and negative effects on university students’ sleep quality (Sanusi et al., 2022; Zhang et al., 2022).

The literature also indicated that the association between mobile phone use and mental health is significantly affected by sleep quality (Xie et al., 2018; Zou et al., 2019; Ho, 2021). This is illustrated in Xie et al.’s (2018) work, which revealed that the association between smartphone overuse and clinical health symptoms is mediated by sleep quality. Similarly, Ho (2021) confirmed the mediating role of sleep quality in the link between addiction to Facebook and depression. To sum up, mobile phone overuse negatively affects university students’ sleep quality, which in turn leads to mental health problems (Zou et al., 2019). Accordingly, we put forward the supposition below:

*Hypothesis 2*: Sleep quality mediates the negative association between mobile phone addiction and mental health.

### 2.3. The moderating effect of perceived social support

Social support refers to “the functions performed for the individual by significant others (including family members, relatives, and friends) and people from extended relationships” (Thoits, 2011, p. 3). Extensive research has confirmed the significant impact of perceived social support on health-related problems, such as psychological well-being and depressive symptoms (Heerde and Hemphill, 2018; Xu et al., 2018; Hirsch et al., 2019; Seon et al., 2019). Previous studies also considered perceived social support as a significant predictor of mental health (Xu et al., 2018; Lee, 2020; Litwiller et al., 2022). In other words, perceived social support is advantageous in maintaining psychological health (Xu et al., 2018; Lee, 2020; Chu et al., 2021). Hu et al. (2020), for instance, asserted that perceived social support can ensure that university students adapt more effectively to independent life, improve their mental health, and protect themselves from risk factors. The findings by Chu et al. (2021) also demonstrated that university students should acquire perceived social support and effective protection from their social networks when they suffer from aggressive bullying.

Given the significant influence of sleep quality on mental health, its interaction with perceived social support may affect university students’ psychological welfare. Previous research has revealed that perceived social support can advance efforts to cope with stress and shield individuals from the consequences of traumatic events (Berg et al., 2021). That is, when social support is perceived as available to them, university students may be less likely to suffer from sleep disorders and a high risk of health-related diseases. In contrast, those with a low level of perceived social support possibly experience poor sleep quality and then run greater risks of suffering from mental illnesses. On the basis of these arguments, we expected university students who perceive having adequate perceived social support to exhibit better sleep quality and thereby enjoy better mental health, as put forward in Hypothesis 3.

*Hypothesis 3*: The negative association between sleep quality and mental health is moderated by perceived social support.

Based on the above-mentioned moderation, we also anticipated that perceived social support may moderate the indirect impact of mobile phone addiction on mental health. Specifically, a high perception of the availability of social support among university students may mitigate the detrimental impact of mobile phone overuse on health-related problems through diminished sleep quality. That is, when university students perceive considerable social support from their families, relatives, and friends, they may experience high levels of sleep quality, which subsequently promotes their mental health. By contrast, university students may suffer from low levels of sleep quality and even worse mental health when they regard perceived social support as missing. In line with such reasoning, we expected the indirect effects of mobile phone addiction on mental health through sleep quality to vary across levels of perceived social support. We therefore posit the following:

*Hypothesis 4*: The mediation effect of mobile phone addiction on mental health through sleep quality is moderated by perceived social support.

## 3. Methods

### 3.1. Participants

We recruited participants from two universities in Yunnan, southern China, after which we administered a cross-sectional survey to five randomly chosen classes in each university from October 2021 to January 2022. We distributed 600 questionnaires and received 592. After excluding incomplete and invalid questionnaires, we obtained a final valid sample of 585 university students (97.5% response rate). Of the participants, 55.9% were male and 44.1% were female, aged between 18 and 25 years (*M*_age_ = 20.33). The participants spent 4.23 h on their mobile phones per day and made or received an average of 3.41 calls per day.

### 3.2. Measures

To ensure the consistency of scales in the present study, we translated validated original scales into Chinese. All items were rated using a five-point Likert scale ranging from 1 (*strongly disagree*) to 5 (*strongly agree*).

#### 3.2.1. Mobile phone addiction

Mobile phone addiction was measured using Leung’s (2008) 17-item scale. Sample items included “You have used your mobile phone to make yourself feel better when you were feeling down” and “Your productivity has decreased as a direct result of the time you spend on the mobile phone.” The Cronbach’s alpha of this scale was 0.959.

#### 3.2.2. Sleep quality

We measured sleep quality adopting a seven-item scale developed by Bastien et al. (2001). Sample items include “How satisfied are you with your current sleep pattern?” and “To what extent do you consider your sleep problem to interfere with your daily functioning?” The Cronbach’s alpha of the scale was 0.806.

#### 3.2.3. Perceived social support

Perceived social support was assessed using a 12-item scale developed by Zimet et al. (1988). Sample items included “I have a special person who is a real source of comfort to me” and “There is a special person in my life who cares about my feelings.” The Cronbach’s alpha in the present study was 0.957.

#### 3.2.4. Mental health

Mental health was measured using the 12-item General Health Questionnaire developed by Gelaye et al. (2015). Sample items are “Enjoy day-to-day activities” and “Feel reasonably happy.” The Cronbach’s alpha of this scale was 0.959.

### 3.3. Data analysis

We first used SPSS version 26 to conduct the descriptive statistical and correlational analyses. As suggested by Hayes and Preacher (2014), we then tested mediating and moderating effects by running the Process macro (version 3.0) for SPSS (model 14). The indirect, moderating, and conditional indirect effects in relation to mobile phone addiction and mental health were analyzed using the biased-corrected bootstrapping method.

## 4. Results

### 4.1. Descriptive statistics and correlations

Table 1 presents the descriptive, correlation, and reliability results with respect to the variables of interest. Mobile phone addiction was positively associated with sleep quality (*r* = 0.872, *p* < 0.01), but it was negatively related to mental health (*r* = −0.886, *p* < 0.01) and perceived social support (*r* = −0.770, *p* < 0.01). Sleep quality was negatively correlated with mental health (*r* = −0.875, *p* < 0.01) and perceived social support (*r* = −0.707, *p* < 0.01). Finally, perceived social support was positively associated with mental health (*r* = 0.740, *p* < 0.01).

**Table 1:** Means, standard deviations and correlations among variables.

Variables	1	2	3	4	5	6	7	8
1. Gender	1							
2. Age	−0.053	1						
3. Time using MP	−0.014	−0.101*	1					
4. Frequency using MP	−0.057	0.058	−0.153**	1				
5. MPA	0.088*	−0.274**	−0.344**	0.133**	(0.959)			
6. SQ	0.098*	−0.226**	−0.387**	0.147**	0.872**	(0.806)		
7. PSS	−0.031	0.332**	0.300**	−0.088*	−0.770**	−0.707**	(0.957)	
8. MH	−0.054	0.253**	0.391**	−0.123**	−0.886**	−0.875**	0.740**	(0.959)
Mean	1.441	20.327	4.226	3.564	3.120	2.585	2.167	2.880
SD	0.497	1.652	2.684	5.125	1.095	0.716	0.818	1.145

*N* = 585. Boldface values indicate Cronbach’s alpha. MP, mobile phone; MPA, mobile phone addiction; SQ, sleep quality; PSS, perceived social support; MH, mental health.

*p < 0.05, **p < 0.01.

### 4.2. Hypothesis testing

Table 2 shows that mobile phone addiction negatively affected mental health (*B* = −0.881, *p* < 0.001), supporting Hypothesis 1. The table also indicates that mobile phone addiction was significantly related to sleep quality (*B* = 0.543, *p* < 0.001) and that sleep quality was significantly related to mental health (*B* = −0.657, *p* < 0.001). Moreover, the bootstrap-derived indirect impact of mobile phone addiction on mental health were significant (*B* = −0.357, 95% confidence interval [CI]: [−0.438, −0.269]). Thus, sleep quality partially mediated the negative association between mobile phone addiction and mental health, supporting Hypothesis 2.

**Table 2:** Results of mediating hypotheses.

Variables	Sleep quality	Mental health	
	Model 1	Model 2	Model 3
Constant	1.014 (0.220)***	4.895 (0.332)***	5.561 (0.305)***
Gender	0.035 (0.029)	0.055 (0.044)	0.078 (0.040)*
Age	−0.004 (0.009)	0.023 (0.014)	0.020 (0.013)
Time using MP	−0.026 (0.006)***	0.045 (0.009)***	0.028 (0.008)***
Frequency using MP	0.003 (0.003)	0.001 (0.004)	0.003 (0.004)
Mobile phone addiction	0.543 (0.015)***	−0.881 (0.022)***	−0.031 (0.002)***
Sleep quality			−0.657 (0.057)***
Total effect [95% CI]		−0.881 [−0.926, −0.837]	
Direct effect [95% CI]		−0.525 [−0.597, −0.453]	
Indirect effect [95% CI]		−0.357 [−0.438, −0.269]	
*R^2^*	0.770***	0.794***	0.833***

Bootstrap size = 5000. CI, confidence interval. *p < 0.05, ***p < 0.001.

**Figure 2:**
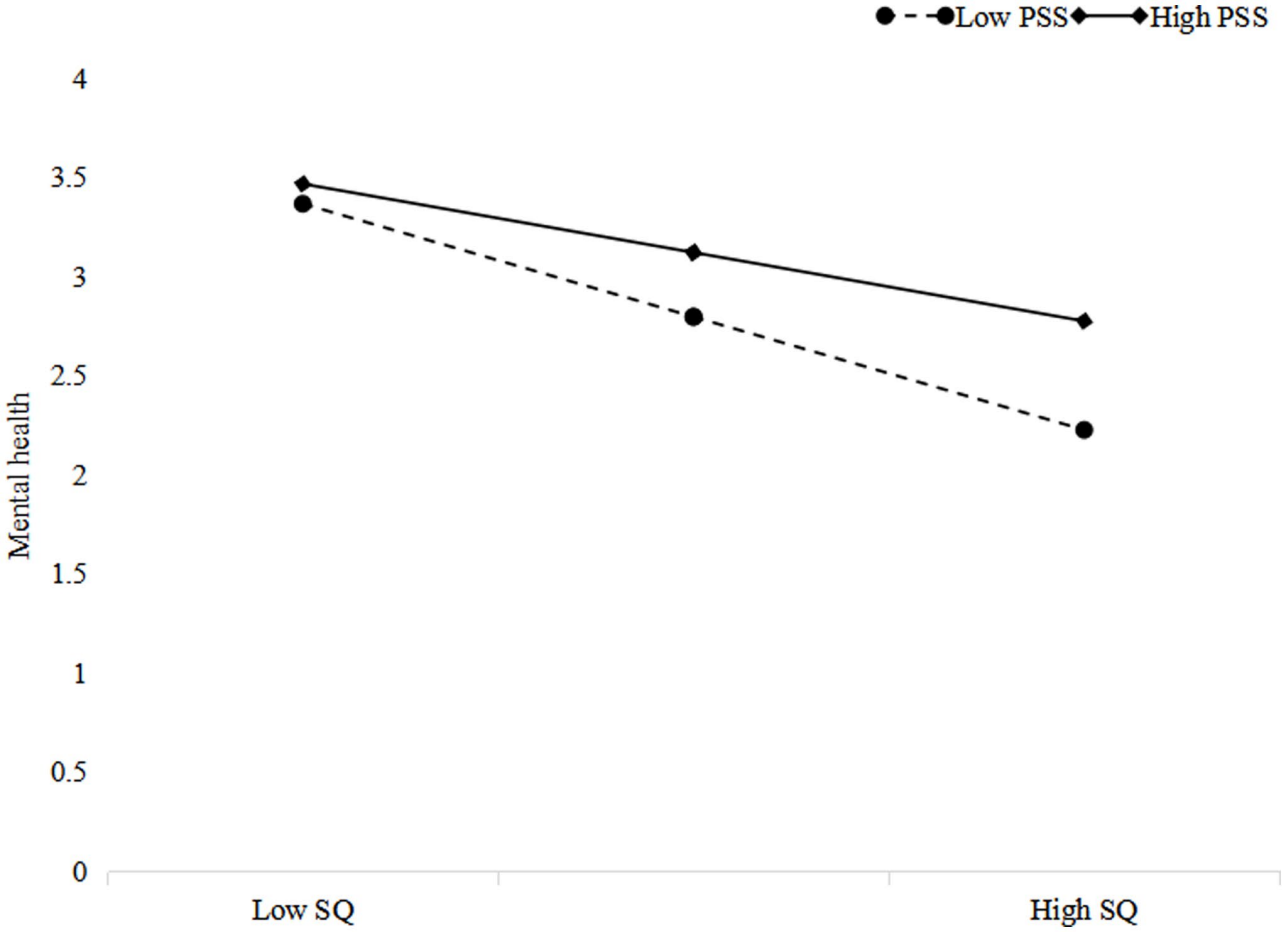
Interaction plot of sleep quality (SQ) and perceived social support (PSS) predicting mental health.

The interaction between sleep quality and perceived social support affected the university students’ mental health significantly and positively (*B* = 0.192, *p* < 0.001; Table 3). We drew an interaction plot (Figure 2) to further explain the results. Figure 2 shows that the effect of sleep quality on mental health was weaker among those students who perceived receiving more perceived social support. This finding supports Hypothesis 3.

**Table 3:** Results of moderating hypotheses.

Variables	*B*	SE	*p*	Boot LLCI	Boot ULCI
Outcome variables: mental health					
Constant	3.854	0.306	0.000	3.253	4.455
Gender	0.072	0.039	0.062	−0.004	0.148
Age	0.014	0.013	0.261	−0.011	0.039
Time using MP	0.012	0.009	0.154	−0.005	0.029
Frequency using MP	0.003	0.004	0.442	−0.005	0.010
Mobile phone addiction (X)	−0.433	0.041	0.000	−0.513	−0.353
Sleep quality (M)	−0.640	0.056	0.000	−0.750	−0.531
Perceived social support (W)	0.199	0.041	0.000	0.117	0.280
M × W	0.192	0.049	0.000	0.097	0.287
*R^2^* = 0.841***, *F*-value = 380.399					

Bootstrap size = 5000. Boot SE, bootstrapping standardized error; LL, low limit; UL, upper limit; CI, confidence interval. ***p < 0.001.

Finally, to test the hypothesized moderated mediation, we determined the conditional indirect impact of mobile phone addiction on mental health through sleep quality (the mean level, one standard deviation above the mean level, and one standard deviation below the mean level). Table 4 reflected that mobile phone addiction had significant indirect effects on mental health across all levels of perceived social support. The indirect effects were significant but weakened as perceptions progressed from low perceived social support (*B* = −0.433, 95% CI: [−0.529, −0.337]) to high perceived social support (*B* = −0.262, 95% CI: [−0.356, −0.163]). These findings are supported by the index of moderated mediation (*B* = 0.095, 95% CI: [0.043, 0.149]), which in turn translates to support for Hypothesis 4.

**Table 4:** Results for the index of moderated mediation and conditional indirect effects.

Moderator: perceived social support	Effect/Index	Boot SE	Boot LLCI	Boot ULCI
Conditional indirect effects [95% CI]				
M – SD (−0.818)	−0.433 (0.049)		−0.529	−0.337
Mean	−0.348 (0.042)		−0.429	−0.264
M + SD (+0.818)	−0.262 (0.049)		−0.356	−0.163
Index of moderated mediation [95% CI]				
Perceived social support	0.104 (0.030)		0.048	0.167

Bootstrap size = 5000. Boot SE, bootstrapping standardized error; LL, low limit; UL, upper limit; CI, confidence interval.

*p < 0.05, **p < 0.01, ***p < 0.001.

## 5. Discussion and conclusion

This study investigated the linkage between mobile phone addiction and mental health, as well as the underlying mechanisms that influence the aforementioned relationship. Our findings are summarized as follows. To begin with, our results indicated that mobile phone addiction affected mental health significantly and negatively. That is, university students who indulge in mobile phones are more vulnerable to psychological disorders. This finding is in line with those of previous empirical studies (Jun, 2016; Desouky and Abu-Zaid, 2020; Ivanova et al., 2020; Ophir et al., 2020; Perilli et al., 2021; Sümen and Evgin, 2021), most of which confirmed a negative relationship between mobile phone addiction and mental health, giving rise to problems such as anxiety, well-being, depression, and other psychological disorders. Thus, our research adds to the empirical evidence on mobile phone addiction leading to mental health problems (Cheng and Meng, 2021).

Second, our study revealed that sleep quality partially mediated the association between mobile phone addiction and mental health. In line with prior studies (Adams et al., 2013; Xie et al., 2018; Zou et al., 2019), the current work confirmed the mediating effect of sleep quality on the link between mobile phone addiction and mental health in the Chinese context. According to technology addiction theory (Chen et al., 2016), technology addiction disrupts university students’ normal sleep patterns, which then poses a high risk to their mental health. The university students with considerable addiction to mobile phones or the internet generally had lower levels of sleep quality, thereby giving rise to depressive symptoms and affective disorders (Lauren et al., 2017; Carlin, 2018). On this basis, the current work strengthens the empirical evidence on sleep quality’s partial mediation of the linkage between mobile phone addiction and mental health among Chinese university students.

Third, perceived social support moderated the association between sleep quality and mental health. To wit, the university students who perceived the availability of substantial social support had better sleep quality, thereby bringing about greater levels of mental health. As can be seen, perceived social support can mitigate the harmful impacts of inferior sleep quality on mental health given that it protects and promotes university students’ psychological well-being (Watson et al., 2016; Fang and Lung, 2022). Although scholars have generally regarded perceived social support as an antecedent of mental health, our research confirmed the moderating effects of such support in the association between sleep quality and mental health for the first time.

### 5.1. Theoretical implications

The present study has made certain theoretical contributions to the literature on mobile phone addiction. First, our findings confirmed the adverse effect of mobile phone addiction on mental health among Chinese university students. Although the direction of causality between mobile phone addiction and mental health remains disputed (Augner et al., 2023), the present study extends scholarship on mobile phone addiction based on a Chinese sample. Given that previous research presented mixed and inconsistent results regarding the linkage between mobile phone addiction and mental health across nations (Jun, 2016; Park et al., 2019; Dowran, 2020), the present research offers new insights into the aforementioned relationship from a cross-cultural perspective. Second, this research uncovered the mechanism that mediates the relationship mobile phone addiction and mental health. To date, only a few empirical studies have focused on the significant effects of sleep quality on mobile phone addiction and health-related issues (Tao et al., 2017; Xie et al., 2018; Zou et al., 2019; Ho, 2021). The majority of these endeavors paid attention to the various directions in which the above-mentioned link occurs (Jun, 2016; Park et al., 2019; Augner et al., 2023), but little attention has been paid to the potential mediators of this relationship. Our study extends existing efforts by proposing sleep quality as a mediator. Third, the research also broadens the application of social support theory by highlighting the protective role of perceived social support for university students with extensive mobile phone addiction. Whereas previous studies primarily considered perceived social support a mediator in research models related to mobile phone addiction (Guo et al., 2021; Lin et al., 2021), the present work is the first to explore the moderating effects of perceived social support in the relationships of mobile phone addiction and sleep quality with mental health. By doing so, our study extends the application and generalization of social support theory to the Chinese context (Wang et al., 2018; Zhou et al., 2021).

### 5.2. Practical implications

Our research also provides some practical implications for students and administrators at Chinese universities. First of all, in view of the significant effects of mobile phone addiction on mental health, mobile phone use can be regarded as a key criterion for assessing levels of self-reported mental health. University administrators should prevent mobile phone addiction among students through various effective and accessible early intervention programs, such as traditional classroom education, keynote speeches, and brochure distribution. For their part, university students should acutely realize that their physical and psychological health will be influenced by mobile phone addiction negatively. A necessary task is for them to focus on real life by reducing mobile phone use. Second, administrators should also realize the urgency of taking effective measures to improve students’ sleep quality. Most sleep-deprived university students suffer from serious mobile phone addictions. Therefore, administrators should distinguish such students from the rest of the student body by conducting a sleep quality epidemiological investigation and then providing effective consultation and intervention, as well as periodically carrying out psychological tests. Additionally, administrators can establish mutual aid dormitory groups to help students get into the habit of going to bed on time. Third, our results stressed the positive effect of perceived social support in mitigating the risk of health-related problems resulting from poor sleep quality. Administrators should encourage students to secure part-time work or provide such employment for them because extracurricular activities potentially increase the perceived social support received by students (Lee, 2020). Because perceived social support can enhance students’ mental health significantly (Xu et al., 2018), university students should have access to increased social assistance from parents, teachers, and peers. Specifically, parents should offer high-quality emotional attention and support to their children. Continuing extensive perceived social support from teachers in school can help university students complete their studies. More importantly, emotional sharing and information acquisition among significant peers can guarantee that university students experience increased understanding, concern, and love.

### 5.3. Limitations and future research directions

Our research is encumbered by several limitations. To begin with, the cross-sectional research design precluded us from clarifying the causal relationships between the variables. Although our findings revealed a negative association between mobile phone addiction and university students’ mental health, the results should be explained with caution. Given that our findings based on a cross-sectional design may be changeable and become uncertain with time, researchers should exploit experimental designs, longitudinal data, or panel data to test causal claims in the future. Second, we collected self-reported data from the respondents, potentially resulting in common method bias, which may drive artificially inflated correlations (Crampton and Wagner, 1994). Hence, other researchers should collect data through different types of surveys. For instance, perceived social support can be ascertained through self-reports from students, while other variables, such as mental health and sleep quality, should be measured by doctors. Third, the study was conducted in one region, southwest China, thus preventing the findings from being generalized to universities in other regions, particularly developed eastern coastal areas. Previous research confirmed the negative impacts of mobile phone addiction on mental health across different groups or regions (Park et al., 2019; Dowran, 2020; Ivanova et al., 2020). Future explorations should therefore look into the factors influencing mobile phone addiction among university students in other regions of China.

## Data availability statement

The original contributions presented in the study are included in the article/supplementary material, further inquiries can be directed to the corresponding author.

## Ethics statement

Ethical review and approval was not required for the study on human participants in accordance with the local legislation and institutional requirements. Written informed consent from the patients/participants was not required to participate in this study in accordance with the national legislation and the institutional requirements.

## Author contributions

L-LY: Conceptualization, Formal analysis, Investigation, Writing – original draft. CG: Conceptualization, Investigation, Writing – original draft, Formal analysis. G-YL: Conceptualization, Formal analysis, Investigation, Writing – original draft. K-PG: Conceptualization, Investigation, Writing – original draft, Project administration, Writing – review & editing. J-HL: Conceptualization, Formal analysis, Investigation, Writing – original draft.
